# Treatment outcomes of patients with adenocarcinoma of the uterine cervix after definitive radiotherapy and the prognostic impact of tumor-infiltrating CD8^+^ lymphocytes in pre-treatment biopsy specimens: a multi-institutional retrospective study

**DOI:** 10.1093/jrr/rrz106

**Published:** 2020-02-13

**Authors:** Yuhei Miyasaka, Yuya Yoshimoto, Kazutoshi Murata, Shin-ei Noda, Ken Ando, Takeshi Ebara, Noriyuki Okonogi, Takuya Kaminuma, Seiji Yamada, Hayato Ikota, Hideaki Yokoo, Tatsuya Ohno, Takashi Nakano

**Affiliations:** 1 Depatment of Radiation Oncology, Gunma University Graduate School of Medicine, 3-39-15, Showa-machi, Maebashi, Gunma 371-8511, Japan; 2 Department of Radiation Oncology, Fukushima Medical University, 1, Hikarigaoka, Fukushima, Fukushima 960-1247, Japan; 3 Department of Radiation Oncology, Saitama Medical University International Medical Center, 1397-1, Yamane, Hidaka, Saitama 350-1241, Japan; 4 Department of Radiation Oncology, Gunma Prefectural Cancer Center, 617-1, Takahayashinishi-cho, Ota, Gunma 373-8550, Japan; 5 Department of Radiation Oncology, Kyorin University, 6-20-2, Shinkawa, Mitaka, Tokyo 181-8611, Japan; 6 National Institute of Radiological Sciences, National Institutes for Quantum and Radiological Science and Technology, 4-9-1 Anagawa, Inage-ku, Chiba 263-8555, Japan; 7 Department of Human Pathology, Gunma University Graduate School of Medicine, 3-39-15, Showa-machi, Maebashi, Gunma 371-8511, Japan; 8 Department of Diagnostic Pathology, Fujita Health University, 1-98, Dengakugakubo, Kutsukake-cho, Toyoake, Aichi 470-1192, Japan; 9 Department of Diagnostic Pathology, Gunma University Hospital, 3-39-15, Showa-machi, Maebashi, Gunma 371-8511, Japan

**Keywords:** uterine cervical cancer, adenocarcinoma, radiotherapy, programmed cell death-ligand 1, CD8

## Abstract

The current study aimed to evaluate the outcomes of patients with adenocarcinoma (AC) of the uterine cervix after definitive radiotherapy (RT) and to evaluate prognostic factors, including immunity-related molecules. A total of 71 patients with AC of the uterine cervix from multiple Japanese institutions were retrospectively analysed. Histological subtypes were diagnosed according to the 2014 World Health Organization classification. All patients underwent definitive RT comprising external beam RT and intracavitary brachytherapy with or without concurrent chemotherapy. Immunohistochemical studies were performed to detect the expression of programmed cell death-ligand 1(PD-L1) and CD8. The 5-year locoregional control (LC), overall survival (OS) and progression-free survival (PFS) rates for all patients were 61.8, 49.7 and 36.1%, respectively. The LC, OS and PFS rates were not significantly different among the histological subtypes. Membranous PD-L1 expression was not significantly associated with prognosis. Patients with CD8-positive tumor-infiltrating lymphocytes (CD8^+^TILs) in the tumor nests had significantly better OS than patients without CD8^+^TILs in the tumor nests (5-year OS: 53.8 vs 23.8%, *P* = 0.038). As expected, the International Federation of Gynecology and Obstetrics (FIGO) stage (2008) III–IVA and maximum tumor diameter > 40 mm were significantly associated with worse prognosis. In summary, the presence of CD8^+^TILs in the tumor nests has the potential to be an independent favorable prognostic factor for patients with AC of the uterine cervix after definitive RT.

## Introduction

Uterine cervical cancer (UCC) is a common form of cancer and major cause of malignancy-related deaths among young women worldwide [1]. Although the incidence of squamous cell carcinoma (SCC), a major pathological type of UCC, has decreased over time, this has coincided with an increase in the incidence of adenocarcinoma (AC) of the uterine cervix. Currently, AC currently accounts for ~20% of all UCC cases [2].

The standard initial treatment options for early-stage UCC include surgery or radiotherapy (RT; combined external beam radiotherapy [EBRT] and intracavitary brachytherapy [ICBT]). Moreover, concurrent cisplatin-based chemoradiotherapy (CCRT) is used to treat more clinically advanced disease [3]. Although the RT regimen for AC has not been standardized, similar treatments are applied to locally advanced AC and SCC [4]. However, some retrospective studies have shown that the prognosis of AC treated with RT alone was worse than that of SCC [5, 6], and a similar trend was observed in patients treated with CCRT [7, 8]. However, a few studies have investigated AC treated with RT, and the treatment outcomes and impacts of clinicopathological variables on prognosis remain to be surveyed [9].

Anti-cancer immune responses are known to affect the survival of patients with different cancer types [10]. For example, CCRT-induced immunogenic tumor cell death was observed in patients with esophageal cancer [11], and RT-induced anti-tumor immunity and its effects on therapeutic efficacy were shown in a mouse model with EL4 lymphoma cells and Lewis lung carcinoma cells [12]. Moreover, dendritic cell and T cell infiltration of the tumor tissues was associated with better survival rates in patients with AC of the uterine cervix who were treated with RT alone [13].

Programmed cell death-1 (PD-1) and its ligand, PD-L1, are immune checkpoint molecules that suppress anti-cancer immunity. The binding of PD-1 to PD-L1 can suppress the proliferation and activity of cytotoxic CD8^+^T cells as part of the response to cancer-associated antigens [14]. Although some researchers have evaluated the expression of immunity-related molecules in patients with UCC [15–18], this topic has not been studied fully, especially in patients treated with RT. Accordingly, we investigated the treatment outcomes and the prognostic significance of clinicopathological variables, including the expression of immunity-related molecules, in patients with AC of the uterine cervix after definitive RT.

## Patients and Methods

### Patient and tumor characteristics

Patients with untreated AC who received definitive RT at Gunma University Hospital, Gunma Prefectural Cancer Center and National Institute of Radiological Sciences Hospital between January 2000 and December 2015 were enrolled in this retrospective study after the protocol was approved by each Institutional Review Board. The trial has been registered in the UMIN Clinical Trials Registry (no. UMIN000027823). Patients whose pre-treatment biopsy samples were not available and those with synchronous malignancies were excluded. A total of 71 patients were analysed, including 38, 17 and 16 from Gunma University Hospital, Gunma Prefectural Cancer Center and National Institute of Radiological Sciences Hospital, respectively. The patient and tumor characteristics are listed in Table 1. The tumor stages were identified using the International Federation of Gynecology and Obstetrics (FIGO) guidelines (2008).

**Table 1:** Patient and tumor characteristics of included patients (*n* = 71)

Characteristics	
Age at diagnosis (years); median (range)	60 (29–88)
FIGO stage (2008)	
IB	8 (11.3%)
II	28 (39.4%)
III	30 (42.3%)
IVA	5 (7.0%)
PeLN	
Negative	39 (54.9%)
Positive	32 (45.1%)
PALN	
Negative	64 (90.1%)
Positive	7 (9.9%)
MTD	
Non-bulky (≤40 mm)	18 (25.3%)
Bulky (>40 mm)	51 (71.8%)
Data not available	2 (2.8%)

PeLN = pelvic lymph node metastasis, PALN = para-aortic lymph node metastasis.

**Fig. 1.**
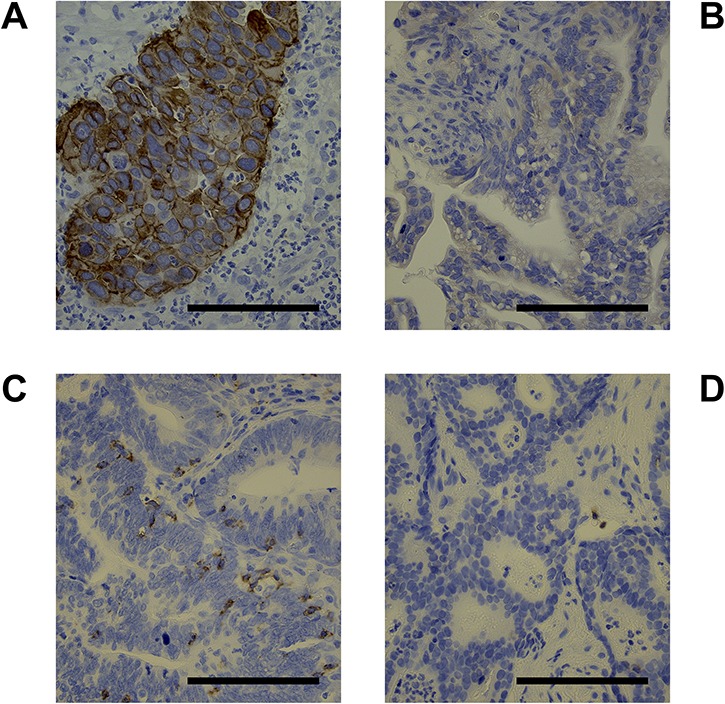
Representative images showing (**A**) positive staining of the tumor cell membrane for programmed death-ligand 1 (PD-L1), (**B**) negative staining for PD-L1, (**C**) positive staining for CD8, and (**D**) negative staining for CD8 (×300). The scale bar is 100 μm.

Pre-treatment formalin-fixed paraffin-embedded biopsy specimens collected from the study patients were sectioned to obtain 4-μm samples, which were stained with hematoxylin and eosin. These samples were then reviewed by two central pathologists who determined the pathological subtypes according to the 2014 World Health Organization (WHO) classification [19]. The pathological diagnoses, including tumor grades and histological subtypes, are shown in Table 2.

### Treatment

All patients underwent definitive RT consisting of EBRT and ICBT. EBRT was delivered at a dose of 1.8 or 2 Gy per fraction, five times per week. A dose of 19.8–40 Gy was delivered to the whole pelvis, followed by pelvic irradiation with a 3-cm-wide central shield. In patients with para-aortic lymph node metastases, the pelvic irradiation fields were extended to include the gross metastatic region. The total dose to the pelvic region was 45–50.4 Gy in 18–28 fractions. For patients with gross lymph node metastases, an additional 6–10 Gy in 3–5 fractions was administered to boost the external dose to the metastases.

**Table 2:** Pathological diagnoses of included patients (*n* = 71)

Subtypes	
Endocervical adenocarcinoma	
Usual type	47 (66.2%)
Mucinous carcinoma	
Gastric type	1 (1.4%)
Signet-ring cell type	1 (1.4%)
Not otherwise specified	6 (8.5%)
Endometrioid carcinoma	1 (1.4%)
Clear cell carcinoma	2 (2.8%)
Serous carcinoma	6 (8.5%)
Adenosquamous carcinoma	7 (9.9%)
Tumor grade	
I	2 (2.8%)
II	31 (43.7%)
III	29 (40.1%)
Data not available	9 (12.7%)

After completing whole-pelvis irradiation, ICBT was performed once per week. EBRT was skipped on the days on which ICBT was performed. ICBT was performed using a low-dose rate (LDR) source in 22.5% of the patients (16/71), a high-dose rate (HDR) source in 76.1% (54/71), and both in 1.4% (1/71). 3D image-guided brachytherapy (3D-IGBT) was administered to 56.3% of the patients (40/71). Briefly, a total dose of 24 Gy was delivered to point A via 4 fractions of HDR-ICBT. Additional ICBT was considered when the tumor response was poor. Interstitial brachytherapy (ISBT) was added along with ICBT when the tumor was bulky and/or asymmetric.

Concurrent chemotherapy was administered to 62.0% of the patients (44/71). Cisplatin alone (40 mg/m^2^), cisplatin (30 mg/m^2^) plus paclitaxel (50 mg/m^2^), and nedaplatin alone (40 mg/m^2^) were administered weekly to 25.3 (18/71), 35.2 (25/71) and 1.4% (1/71) of the patients, respectively.

### Follow-up and assessment of clinical outcomes

The patients were followed up by gynecological oncologists and radiation oncologists every 1–3 months for the first 2 years and every 3–6 months for 3 subsequent years. During each follow-up examination, the disease status was assessed in terms of the locoregional control (LC), overall survival (OS) and progression-free survival (PFS). LC was defined as no evidence of tumor regrowth or recurrence in the pelvic region.

### Immunohistochemical analysis

Immunohistochemical studies were performed to detect the expression of PD-L1 and CD8 in biopsy samples excised from the cervical tumors before RT. Paraffin sections with a thickness of 4 μm were dewaxed in xylene and rehydrated through a graded ethanol series. Endogenous peroxidase activity was blocked by a 10-min incubation in 0.3% hydrogen peroxide. After pretreatments according to the manufacturer’s instructions, the sections were incubated overnight with primary antibodies at 4°C. A commercially available biotin–streptavidin immunoperoxidase kit (Histofine, Nichirei, Tokyo, Japan) and diaminobenzidine were used for coloration. The following antibodies were used: rabbit monoclonal anti-PD-L1 (1:100 dilution, E1L3N; Cell Signaling Technology, Danvers, MA, USA) and mouse monoclonal anti-CD8 (1:200 dilution, M7103; Dako, Carpinteria, CA, USA).

Positive staining for PD-L1 was defined as partial or complete staining of the membranes of viable tumor cells. Immune cells, normal cells, necrotic cells and debris were excluded. PD-L1 positivity was defined as > 1% positively stained tumor cells. CD8 positivity was defined as at least one positively stained lymphocyte in a tumor nest in the biopsy specimen. CD8 negativity was defined as stained lymphocytes only in the stroma (i.e. no stained lymphocytes in the nest). Representative images are shown in Fig. 1.

### Statistical analysis

The LC, OS and PFS rates were calculated using the Kaplan–Meier method. Univariate and multivariate analyses were performed using a Cox proportional hazard model. *P* values <0.05 were considered statistically significant for all tests. All statistical analyses were conducted using SPSS 24.0 for Mac (SPSS, Chicago, IL, USA).

## Results

### Clinical outcomes

**Fig. 2.**
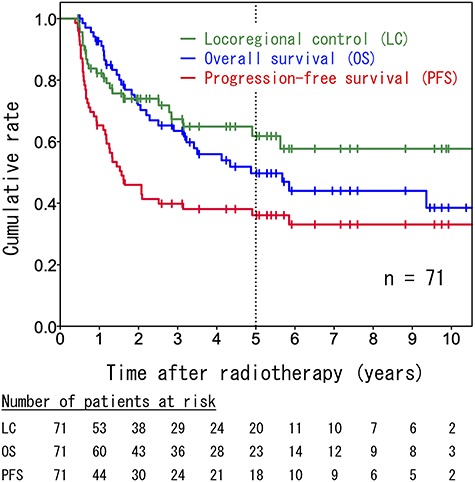
Kaplan–Meier survival curves including all patients (*n* = 71).

**Fig. 3.**
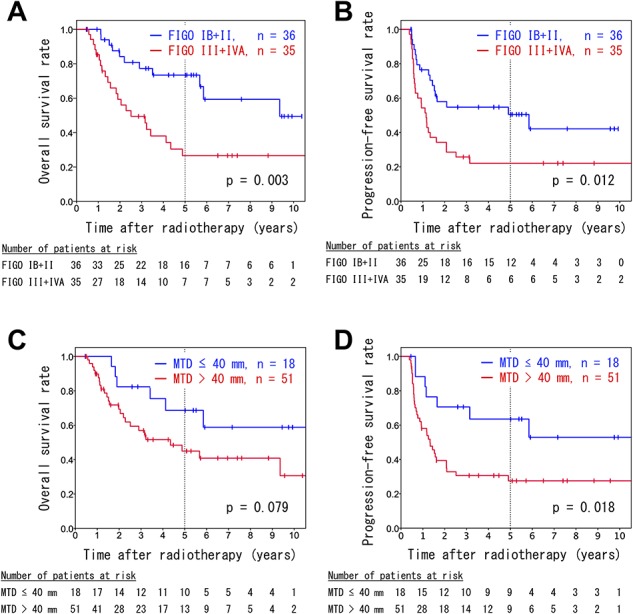
Kaplan–Meier survival curves of analyses stratified by FIGO stage (**A**, **B**) and maximum tumor diameter (**C**, **D**). The overall survival rates (A, C) and progression-free survival rates (B, D) are shown.

The median follow-up durations were 37 months (range, 5–194months) for all patients and 60 months (range, 5–194 months) for surviving patients. The 5-year LC, OS and PFS rates for all patients were 61.8% (95% confidence interval [CI]: 48.5–75.1%), 49.7% (95% CI: 36.6–62.8%) and 36.1% (95% CI: 24.3–47.9%), respectively (Fig. 2). Patients with FIGO stage IB–II disease had significantly better OS and PFS rates than patients with FIGO III–IVA disease (5-year OS: 73.4 vs 26.6%, *P* = 0.003; 5-year PFS: 50.5 vs 22.0%, *P* = 0.012; Fig. 3A and B). Compared to a maximum tumor diameter (MTD) ≤ 40 mm, a MTD > 40 mm tended to be associated with a worse OS; however, this difference did not reach statistical significance (44.9 vs 68.6%, *P* = 0.079; Fig. 3C). However, a MTD > 40 mm was associated significantly with a worse PFS (27.6 vs 63.5%, *P* = 0.018; Fig. 3D).

**Fig. 4.**
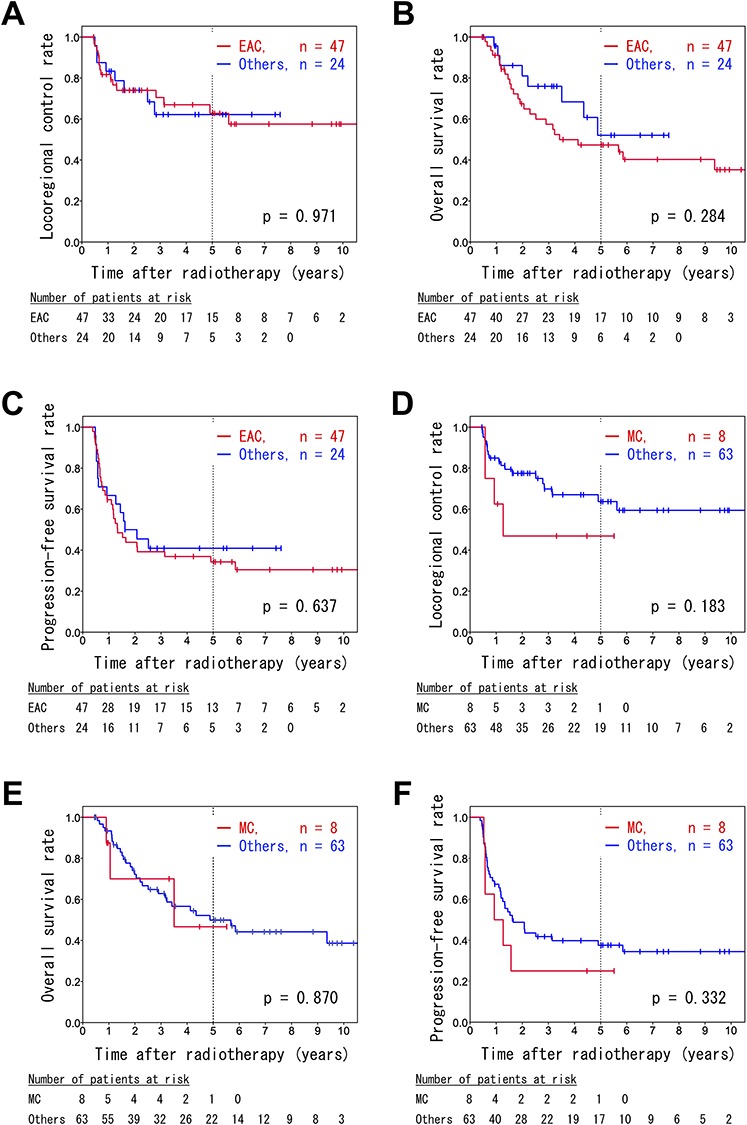
Kaplan–Meier survival curves of patients with endocervical adenocarcinoma, usual type (EAC) (**A**, **B**, **C**) and mucinous carcinoma (**D**, **E**, **F**). The locoregional control (A, D), overall survival (B, E), and progression-free survival rates (C, F) are shown.

We also investigated differences in prognoses among the pathological subtypes. There were no significant differences in the LC, OS and PFS rates between patients with endocervical adenocarcinoma, usual type (EAC), the most common subtype, and the other subtypes (Fig. 4A, B and C). Mucinous carcinoma (MC; including gastric type, signet-ring cell type and not otherwise specified [NOS]) tended to be associated with worse LC when compared to the other subtypes (5-year LC: 46.9 vs 73.0%, *P* = 0.183; Fig. 4D). However, no differences were observed in the OS and PFS rates (Fig. 4E and F). Moreover, the prognoses associated with serous carcinoma and adenosquamous carcinoma did not differ significantly from those associated with the other histological types.

LC rates according to the source of ICBT were analysed, however, there was no significant difference between the LC rates for LDR-ICBT (including mixed-source) vs those for HDR-ICBT (5-year LC: 68.9 vs 72.5%, *P* = 0.370).

### Immunohistochemistry

**Fig. 5.**
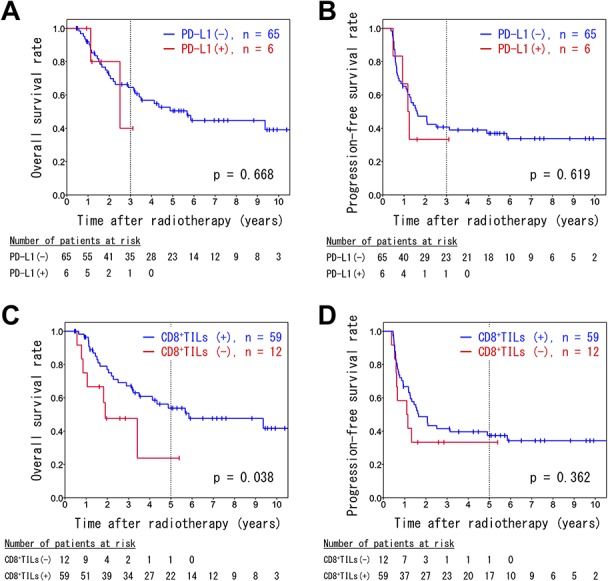
Kaplan–Meier survival curves of analyses stratified by PD-L1 positivity (**A**, **B**) and CD8 positivity (**C**, **D**). The overall survival rates (A, C) and progression-free survival rates (B, D) are shown.

In an immunohistochemical analysis, 8.5% of patients (6/71) exhibited PD-L1 expression on tumor cell membranes. A log-rank analysis did not reveal any significant differences in OS and PFS between patients with and without membranous PD-L1-positive tumors (3-year OS: 64.5% [95% CI: 52.2–76.8%] vs 40.0% [95% CI: 0–98.2%], *P* = 0.668; 3-year PFS: 40.7% [95% CI: 28.5–52.9%] vs 33.3% [95% CI: 0–70.9%], *P* = 0.619; Fig. 5A and B).

**Table 3:** Univariate and multivariate analyses of overall survival

		Overall survival					
		Univariate		Multivariate (A)		Multivariate (B)	
Factor	Category	HR	*P*	HR	*P*	HR	*P*
		95% CI		95% CI		95% CI
Age (years)	<60	1.39	0.348	1.43	0.395		
	≥60	0.70–2.77		0.63–3.28		
FIGO stage	IB + II	2.91	0.004	4.49	0.001	3.62	0.002
	III + IVA	1.41–6.03		1.91–10.57		1.63–8.02
MTD (mm)	≤40	2.19	0.086	7.59	0.003	6.06	0.003
	>40	0.89–5.36		2.02–28.56		1.88–19.49
PeLN	Negative	1.10	0.793	1.46	0.450		
	Positive	0.54–2.22		0.55–3.91		
Tumor grade	I + II	1.12	0.752	0.50	0.119		
	III	0.55–2.30		0.21–1.20		
PD-L1	Negative	1.37	0.670	0.36	0.352		
	Positive	0.32–5.90		0.04–3.13		
CD8^+^TILs	Negative	0.41	0.044	0.13	0.003	0.17	0.002
	Positive	0.17–0.98		0.04–0.49		0.06–0.52
Concurrent chemotherapy	No	0.67	0.266	0.32	0.024	0.43	0.043
	Yes	0.33–1.36		0.12–0.86		0.19–0.97

HR = hazard ratio, PeLN = pelvic lymph node metastasis.

**Table 4:** Univariate and multivariate analyses of progression-free survival

		Progression-free survival					
		Univariate		Multivariate (A)		Multivariate (B)	
Factor	Category	HR	*P*	HR	*P*	HR	*P*
		95% CI		95% CI		95% CI	
Age (years)	<60	0.97	0.967	1.05	0.902		
	≥60	0.55–1.79		0.52–2.12		
FIGO stage	IB + II	2.14	0.014	2.56	0.006	2.28	0.012
	III + IVA	1.16–3.94		1.30–5.05		1.20–4.32
MTD (mm)	≤40	2.58	0.023	3.00	0.039	3.27	0.009
	>40	1.14–5.83		1.06–8.53		1.34–7.98
PeLN	Negative	1.40	0.268	1.49	0.335		
	Positive	0.77–2.54		0.66–3.33		
Tumor grade	I + II	1.26	0.475	0.80	0.556		
	III	0.67–2.35		0.39–1.67		
PD-L1	Negative	1.30	0.621	0.62	0.497		
	Positive	0.46–3.66		0.16–2.45		
CD8^+^TILs	Negative	0.70	0.366	0.37	0.053	0.39	0.032
	Positive	0.32–1.51		0.13–1.01		0.16–0.92
Concurrent chemotherapy	No	1.15	0.671	0.85	0.698		
	Yes	0.61–2.14		0.38–1.92		

HR = hazard ratio, PeLN = pelvic lymph node metastasis.

Tumor-infiltrating lymphocytes positive for CD8 (CD8^+^TILs) in the tumor nests were observed in 83.1% of the patient biopsy samples (59/71). Patients with CD8^+^TILs had a better OS rate compared to patients without CD8^+^TILs (5-year OS: 53.8% [95% CI: 17.7–67.9%] vs 23.8% [95% CI: 0–59.9%], *P* = 0.038; Fig. 5C). There was no significant difference in the PFS rate between patients with and without CD8^+^TILs (5-year PFS: 37.4% [95% CI: 24.6–50.1%] vs 33.3% [95% CI: 6.6–60.0%], *P* = 0.362; Fig. 5D).

### Prognostic factors

The OS and PFS outcomes according to age, FIGO stage, MTD, pelvic lymph node status, tumor grade and expression of PD-L1 and CD8 were analysed using a Cox proportional hazard model (Tables 3 and 4). No strong correlations (0.5 > | *r* |) were observed among these factors. Univariate analyses revealed that a FIGO stage III–IVA was significantly correlated with an unfavorable OS (*P* = 0.004) and PFS (*P* = 0.014), while a MTD > 40 mm was a significant predictor of an unfavorable PFS (*P* = 0.023) and the presence of CD8^+^TILs was a favorable prognostic factor for OS (*P* = 0.044).

In multivariate analysis (A) including all the above-mentioned factors, FIGO stage III–IVA and a MTD > 40 mm were correlated with a worse OS (*P* = 0.001 and 0.003, respectively) and PFS (*P* = 0.006 and 0.039, respectively). In contrast, the presence of CD8^+^TILs was associated with a better OS (*P* = 0.003) and tended to be correlated with a better PFS (*P* = 0.053). The administration of concurrent chemotherapy was also a favorable predictor of OS (*P* = 0.024). Multivariate analysis (B) was then performed with the inclusion of factors with *P* values < 0.1 in multivariate analysis (A). Multivariate analysis (B) revealed that the presence of CD8^+^TILs was a significant prognostic factor for both OS and PFS (*P* = 0.002 and 0.032, respectively) in addition to the FIGO stage and MTD.

## Discussion

In the present study, we evaluated 71 patients with AC of the cervix who were treated with definitive RT and analysed the prognostic significance of clinicopathological variables. Regarding the pathological subtypes, the most common subtype, EAC, was not correlated with a better or worse LC, OS or PFS. In addition, MC, including gastric type, signet-ring cell type and NOS, were not significant predictors of LC, OS and PFS. MC, gastric type, an aggressive tumor type with gastric pyloric differentiation, accounts for ~30% of all cases of AC in Japanese patients [20–22]. However, only 1 patient (1.4%) in the current study had MC, gastric type. This difference may be attributable to the greater likelihood that patients with MC would have undergone surgery, which was associated with a better prognosis relative to RT in this patient population [23].

The histological grade did not have a significant prognostic effect in the current study, although no recurrence or metastasis was observed in 2 patients with grade 1 disease. In contrast, another study identified the histological grade as a significant prognostic factor [24]. Notably, 66% of patients in the previous study were treated surgically, and the patient characteristics differed between the studies. Accordingly, further studies are required to settle the controversy surrounding the prognostic effect of histological grade in patients treated with RT [9].

It was previously reported that LDR- and mixed-source- ICBT yielded better LC compared with that for HDR-ICBT [25]. In the current study, however, no significant difference in LC rates according to the kind of source of ICBT was observed.

Previous research has identified negative correlations between PD-L1 expression and prognosis in patients with several kind of malignancies [26]. Moreover, a randomized controlled trial reported that chemoradiotherapy followed by a PD-L1 inhibitor yielded prognostic benefits in patients with locally advanced non-small cell lung cancer [27]. Therefore, we assumed that PD-L1 expression could lead to a poor prognosis in patients treated with RT and evaluated PD-L1 expression on the tumor cell membranes. Although we observed membranous PD-L1 expression in 8.5% of the patients (6/71), Heeren *et al*. reported that 10–17% of cases of AC of the cervix exhibited PD-L1 positivity [18]. We note that tumor characteristics might be responsible for differences in positivity rates, as more advanced cases tend to exhibit PD-L1 positivity on tumor membranes [28]. In the present study, we did not identify a prognostic significance of PD-L1. However, one previous study reported PD-L1 upregulation after X-ray exposure [29]. Therefore, a future investigation of PD-L1 expression in post-treatment biopsy samples may reveal prognostic significance.

We identified a significant correlation of the presence of CD8^+^TILs in the tumor nest with better prognoses in patients with AC. Previously, Jordanova *et al*. surveyed intraepithelial TILs in surgically treated UCCs and identified the CD8^+^/regulatory T cell ratio as a significantly unfavorable prognostic factor [30]. Komdeur *et al*. reported an association of the presence of tumor-reactive intraepithelial CD8^+^T cells with CD103 expression and identified the prognostic impact of CD103 expression in patients who received RT [31]. Enwere *et al*. reported a trend toward a favorable PFS after CCRT for UCC in patients whose tumors contained CD8^+^TILs (*P* = 0.120). This trend was more noteworthy in patients with tumors positive for PD-L1 (*P* = 0.052) [15]. In patients with AC who were treated with RT alone, Nakano *et al*. performed immunohistochemistry for S-100 and CD43 and identified a positive correlation between dendritic cell and T cell infiltration, as well as the prognostic significance of immunologic cell infiltration in tumor tissues. In that study, the 10-year survival rates of patients with and without T cell infiltration were 50 and 30%, respectively (*P* < 0.1) [13]. In summary, these previous studies suggest that tumor-reactive T cell infiltration of the tumor tissues leads to a favorable prognosis, consistent with our results.

Practically, CD8^+^TILs in the tumor nests, as a prognostically significant factor, could be used to determine the subgroups that require careful follow-up. In addition, our results support further consideration of the addition of adjuvant immunotherapy to RT for AC. Currently, many clinical trials are investigating the effect of RT combined with immunotherapy for various types of malignancies [32]. Therefore, a combination of RT with immunotherapy might improve the prognosis of patients with AC and CD8^+^TILs in the tumor nests.

This study had some limitations. As this was a retrospective study of a relatively small number of patients, considered to be an inhomogenous population in many aspects, we could not exclude some potential sources of bias. Moreover, the number of clinicopathological factors included in the multivariate analyses was limited. Our results should be validated in a larger cohort study that also analyses additional influential factors.

In summary, the presence of CD8^+^TILs in the tumor nests was significantly correlated with favorable prognosis in patients with AC treated with definitive RT. Although prospective validation studies are needed, the presence of CD8^+^TILs has the potential to be an independent favorable prognostic factor. Furthermore, it may help to identify the subgroup of patients requiring careful follow-up and may support further exploration of novel treatment strategies, including the combination of RT with immunotherapy.

## Funding

This work was supported by a Grant-in-Aid from the Japan Society for the Promotion of Science (JSPS) for Scientific Research (C; Grant number 17 K10469) and Grants-in-Aid from the Ministry of Education, Culture, Sports, Science, and Technology of Japan for programs for Leading Graduate Schools, “Cultivating Global Leaders in Heavy Ion Therapeutics and Engineering”.

## Conflict of Interest

None declared.
